# The Spatial Patterns of Red Beds and Danxia Landforms: Implication for the formation factors–China

**DOI:** 10.1038/s41598-018-37238-7

**Published:** 2019-02-13

**Authors:** Luobin Yan, Hua Peng, Shaoyun Zhang, Ruoxi Zhang, Milica Kašanin-Grubin, Kairong Lin, Xinjun Tu

**Affiliations:** 1 School of Geographical Sciences, Southwest University, Chongqing, China; 2 School of Geography and Planning, Sun Yat-sen University, Guangzhou, Guangdong China; 3 University of Belgrade, Institute of Chemistry, Technology and Metallurgy, Njegoševa 12, Belgrade, 11000 Serbia

## Abstract

This research examined the distribution features of red beds and 1,100 Danxia landform sites across China, while probing the relationship between these spatial patterns and geological elements. This study is based on geological and tectonic maps of China. ArcGIS software was used to process the adjacent index, then perform a spatial analysis of Danxia landforms and red beds, and a coupling analysis of Danxia landforms and red beds with tectonics. Based on a point pattern analysis of Danxia landforms, the adjacent index is 0.31, and the coefficient of variation verified by Thiessen polygon reaches 449%. These figures reflect the clustered distribution pattern of the Danxia landforms. Across the country, Danxia landforms are concentrated into three areas, namely, the Southeast China region, the Sichuan Basin region and the Qilian-Liupan region. The exposure of red beds covers 9.16 × 10^5^ km^2^, which accounts for 9.5% of the total land area of China. With this research background, the geological elements of tectonics and their effects on the distribution, number, and spatial pattern of Danxia landforms and red beds were analyzed.

## Introduction

Danxia is the name for a unique type of landform and a natural landscape. Literally in Chinese, Danxia means “reddish rays” or “rosy cloud” during a sunrise or sunset. In Chinese geoscience circles, the term “Danxia” was initially designated by geologists in the 1920s to classify a specific stratigraphic sequence of red beds that forms hoodoo peaks in the mountainous terrain of Mt. Danxiashan in southern China. Danxia landform refers to an erosional landscape developed on red beds, characterized by scarp slopes^1^. The study of the Danxia landform began as descriptions and classifications of features, and has evolved into using experimental and quantitative research methods^2,3^.

Being one of the most important tourism attractions in China, Danxia landform plays a significant role in the development of local economy. Besides tourism development, this resource is also beneficial for society and the environment. With the ever increasing tourists, the value of Danxia landform as a scenic resource has become more and more apparent. To better utilize and protect Danxia landform, studying its distribution on a national scale has become unavoidable.

The spatial distribution of Danxia landform is not random. Rather, it is due to this landform being formed under geologically-specific, long-term natural processes. The study of the spatial patterns of Danxia landform shows that its development is not the result of a single force, but rather the consequence of regional geological conditions and geological history. Because red beds are the foundation for the development of Danxia landforms, it is necessary to perform for in depth studies on these red beds^1^.

Red beds are sedimentary rocks typically consisting of conglomerate, sandy conglomerate, sandstone, siltstone, shale and mudstone that are predominantly red in color due to the presence of ferric oxides^4^. Owing to differences in depositional environments and influences of late stage geologic processes, the color of red beds can be brownish-reddish-yellow, brownish-yellow, purplish-red, brownish-red, grayish-purple and other reddish tints. Red beds that are the foundation for Danxia landform are mostly nonmarine. Continental red beds comprise a wide range of sedimentary facies, representing the whole spectrum of non-marine depositional environments: alluvial fans, river floodplains, deserts, lakes and deltas^4^. Although previous studies have suggested that red bed sediments in SE China are related to hot and arid climate conditions during deposition^5–8^, the occurrence of hematite is of no paleoclimatic significance because red beds are known to form in both arid and moist tropical climates^4,9,10^, even a possible thermal origin^11^.

Land degradation in red bed landscapes is a serious environmental and socioeconomic problem in present day China^12^ due to its dominant purple soil texture (Calcaric Regosols in FAO taxonomy)^13^. The knowledge of spatial distribution of red beds is the most basic work for evaluating the land degradation in China. However, the research about the distribution of red beds is relatively weak. In previous research, although the preliminary features of distribution were summarized by some researchers, for example, Zeng Zhaoxuan and Pan Zhixin summarized the general distribution of red beds in southeast China^14^ and in China, respectively^15^, Li Tingyong divided four main red bed regions, southwest China, south China, central China and northwest China^16^. However, all of the above mentioned research is lacking in quantitative methodologies. Moreover, previous research has never discussed the red beds and original distribution.

Overall, the goals of this study are to examine the spatial distribution of red beds and Danxia landforms, and explore the implication of tectonics on their distribution. The spatial distribution features of red beds and Danxia landforms was examined by the Adjacent index method, and their impacting factors were numerically analyzed by spatial overlay analysis and coupling analysis. In addition, the impact of crustal movement on the distribution of red beds and Danxia landforms was discussed under a long-term geodynamic context.

## Material and Methods

### Data sources

Information regarding the distribution, age of red beds and important faults was collected from the National Geological Map (1:1,000,000). 1,100 geographical positions of Danxia sites were collected by Prof. Huang Jin from Sun Yat-sen University who investigated more than 990 Danxia sites in the field in China. According to descriptive geographical position reports, we were able to determine the geographical coordinates from the internet. The outlines of basins formed by different stages of crustal movement were extracted from the National Geotectonic Map produced by the Chinese Academy of Geological Sciences. Other data sources include the National Plate Tectonics Map (based on Plate Tectonic Theory) (1:3,200,000) and the National Tectonics Map (based on Polycyclic Theory) (1:3,200,000) in the book *Geographic Atlas of China*^17^.

### Methods

#### The adjacent analysis

First proposed in 1954 by two ecologists, the adjacent analysis was designed to analyze point patterns in space^18^, and distances to the nearest neighbor as a measure of spatial relationships in populations. It has gradually been developed as a mature method for measuring point distribution patterns. The adjacent analysis model was modified by Pinder^19^ and Ebdon^20^ to assess a variety of dataset distribution patterns, such as geologic hazards^21^, settlement distribution^22^, species spatial distribution^23^, and traffic accident distribution^24^. The modified adjacent analysis model overcomes the terrain restrictions by fitting the study area to a square shape. The closer the study area is to a square box, the greater the accuracy of the adjacent ratio. The coordinate points analyzed in this study were based on the coordinate locations of the Danxia landforms. The Elipse Lambert Azimuthal Equal Area coordinate system was used to calculate the point density.

On the macro level, Danxia sites are dotted throughout China. The nearest neighbor index is the most common method for determining the spatial distribution of point features. The spatial distribution characteristics of Danxia landforms across the country were analyzed using the nearest neighbor index R.

Its equation is^25,26^:

1 $$R=\frac{\overline{{r}_{1}}}{\overline{{r}_{E}}}=\frac{1}{n}\sum _{n}^{1}{r}_{1}({S}_{i})\times (\frac{1}{2\sqrt{\frac{n}{A}}})$$

Where ${\bar{r}}_{1}$ is the average value of the adjacent point to the distance *r*_1_. ${\bar{r}}_{E}$ is the average value of the adjacent point to the distance in the random distribution theory, n is the point number, *r*_1_ (*S*_*i*_) is the distance between spot *S*_*i*_ in the area to the adjacent point, and A is the area. When R = 1, it means the point distribution is random; when R > 1, point distribution is even; when R < 1, point distribution is clustered.

#### Thiessen polygons

There are different definitions and standards for measuring the spatial distribution of points using the adjacent index. This study applied the Thiessen polygon method, which is one method for selecting regional discrete sampling points. The coefficient of variation was defined as the ratio of standard deviation and average of the Voronoi polygon area. It is capable of measuring the variation of relative changes in space. The equation is:

2 $${\rm{CV}}={\rm{S}}/{\rm{M}}$$

Where S is the standard deviation value of the Voronoi polygon area, and M is the average value of the Voronoi polygon area.

#### Spatial analysis method

With the use of GIS, an overlay analysis, a buffer analysis and other related analyses were carried out to discuss the impact of faults and different stages of crustal movement on the distribution of red beds. Firstly, the overlay analysis of red bed distributions and faults was carried out and buffer zones of 0–60 km at 5 km intervals were set to discuss the impact of faults on the distribution of red beds and Danxia landforms. Secondly, the overlay analysis of the red beds distribution and basins formed in different stages of crustal movement was carried out to discuss the impact of crustal movement on the spatial pattern of red beds in different geologic times.

All the analyses discussed above were carried out by ArcGIS 10.2^27^.

## Results and Analysis

### Spatial pattern of red beds

**Figure 1:**
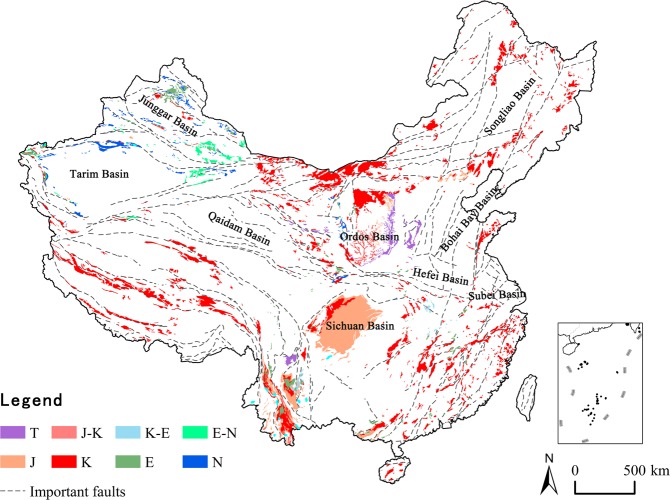
The distribution of red beds in China. The red beds distribution and important faults were identified from 77 National Geological maps (1: 1,000,000), and then the red beds layer was created on ArcGIS 10.2 platform (http://www.esri.com/software/arcgis/arcgis-for-desktop). T-Triassic, J-Jurassic, K-Cretaceous, E-Paleogene, N-Neogene.

**Figure 2:**
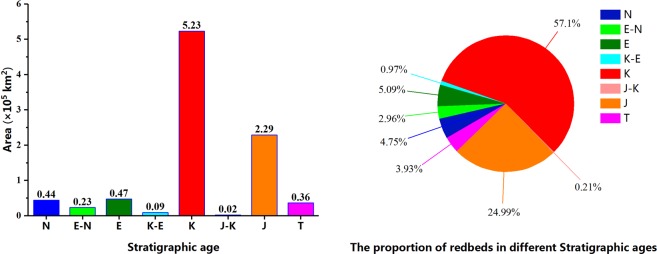
Area and proportions of red beds in different times. T-Triassic, J-Jurassic, K-Cretaceous, E-Paleogene, N-Neogene.

The distribution map of red beds (Fig. 1) was made based off of the combination of the National Geological Map and field investigations, and the area of red beds in different geological ages was measured by ArcGIS. Exposed red beds cover 9.16 × 10^5^ km^2^, which accounts for 9.5% of the total land area of China. Almost all of the red beds in China were deposited during the time spanning from the Triassic period to the Neogene period. Results from previous research show that a small area of red beds were formed prior to the Mesozoic Era, in the Tarim Platform in China, continental coarse classic rocks are also found in the Devonian and Permian Systems^28^. However, due to their small area^29^ and marine facies, we will not discuss these formations in this paper. About 57% of the red beds in China were deposited in the Cretaceous period, 25% in the Jurassic period, 4.8% in the Neogene period, 5.1% in the Paleogene, and 4% in the Triassic period (Fig. 2).

The Triassic red beds are mainly distributed in the east of Ordos Basin (Loess Plateau) and the border of Yanyuan County, Sichuan Province and Ninglang Yi Autonomous County, Yunnan Province. Due to being covered by loess, Triassic red beds are only exposed in strong, cutting gullies with dendritic distributions. Besides, a small proportion in the east and middle of Yunnan Province and the middle of Ordos Basin, the Jurassic red beds are mainly distributed in Sichuan Basin. The Cretaceous red beds have the widest distribution area and can be found across the entire country, but they are found much less in the Xinjiang Uygur Autonomous Region. A scattered distribution along the important faults is the typical feature of Cretaceous red beds. The Paleogene and Neogene red beds are mainly distributed in the margin of Tarim Basin and Junggar Basin, Xinjiang Uygur Autonomous Region.

**Figure 3:**
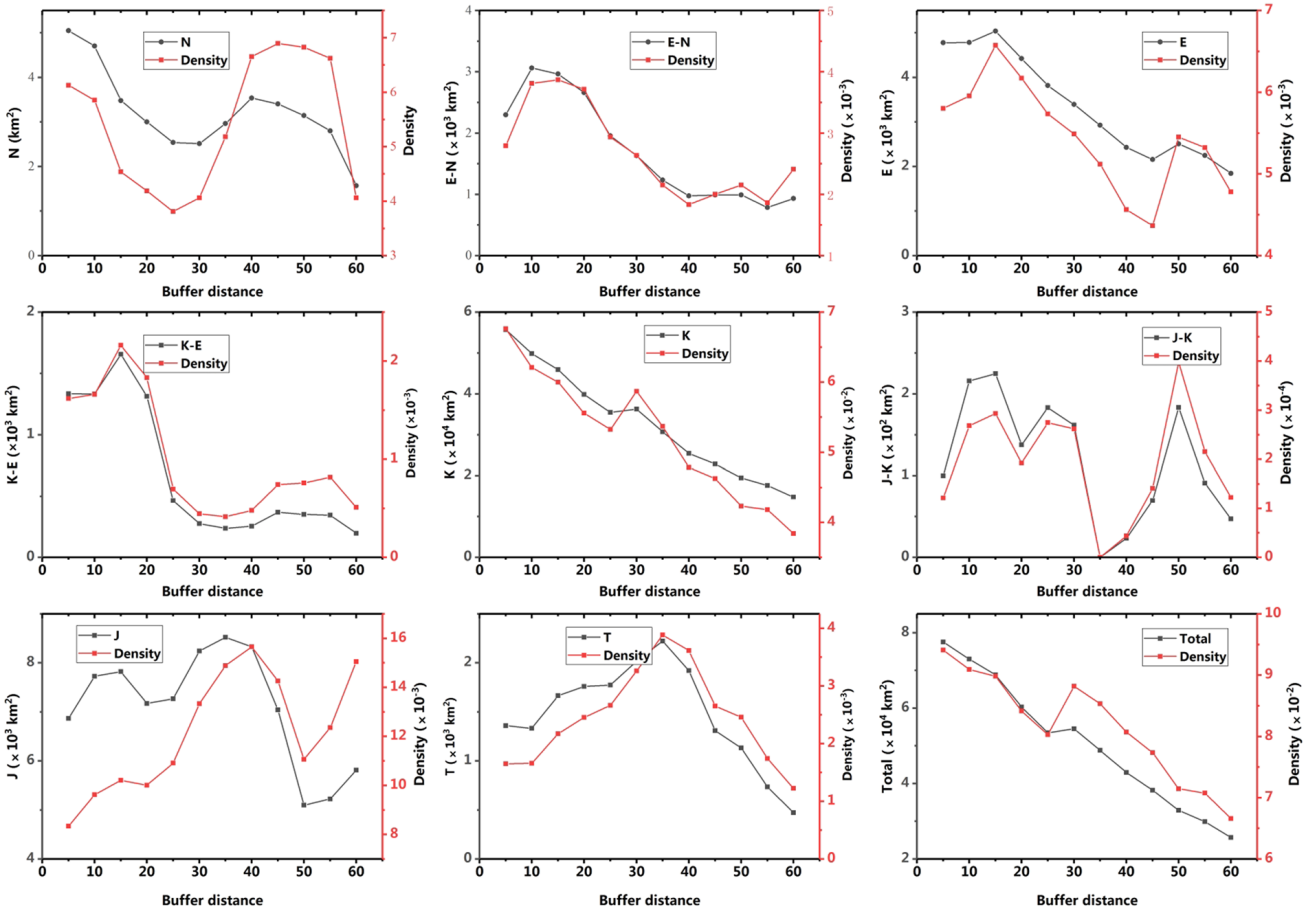
The distribution of red beds in different buffer zones, T-Triassic, J-Jurassic, K-Cretaceous, E-Paleogene, N-Neogene.

The overlay analysis using ArcGIS shows that the areas and proportions of Paleogene and Cretaceous red beds have a negative correlation with the red bed’s distance from a fault (Fig. 3). Applying the linear regression analysis to red beds deposited in different geological ages, for Paleogene and Cretaceous red beds, the regression equations are y = 5.56–0.0731× (Coefficient 0.98) and y = 5.38–0.06× (Coefficient 0.92), respectively, where y is the buffer zones’ areas and x is the buffer’s distance to faults. We can conclude that the formation of faults is an important influential factor for deposition of the Cretaceous red beds.

### Spatial distribution of Danxia landforms

The analysis of Danxia landform distribution was performed by ArcGIS 10.2 software using the adjacent analysis model in function 1. Using China as the study object and EUCLIDEAN DISTANCE as the method, where ${\bar{r}}_{1}$ ≈ 20.59 km and ${\bar{r}}_{E}$ ≈ 66.69 km, the adjacent index is R ≈ 0.31 < 1. The analysis indicates that the spatial distribution of Danxia landforms is highly clustered.

Thiessen polygon maps oriented by the distribution of Danxia landforms points across the country indicate that S ≈ 99455 km^2^, M ≈ 22164 km^2^, and the coefficient of variation CV ≈ 449%. The study by Duyckaerts^30^ shows that the coefficient of variation equals to 33–64% when points are distributed randomly; greater than 64% when points are distributed in clusters; and less than 33% when points are distributed evenly^31^. The results indicate that the Danxia landforms in China are distributed in extremely clusters.

**Figure 4:**
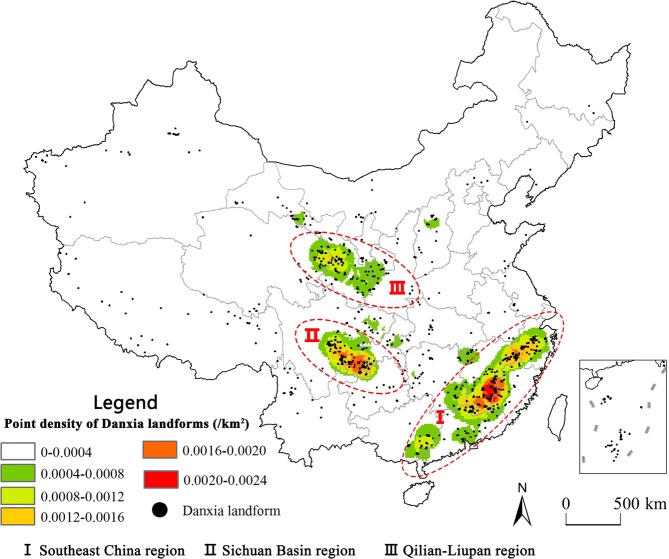
Concentrated Danxia landforms in China. Positions of Danxia landforms supported by Prof. Huangjin, from Sun Yat-sen University, this map was produced by authors on software ArcGIS 10.2 (http://www.esri.com/software/arcgis/arcgis-for-desktop).

Further analysis of the density distribution of Danxia sites (Fig. 4) shows that the distribution of Danxia landforms is patchy. According to the result of point density, three zones were identified: Southeast China region, Sichuan Basin region and Qilian-Liupan region (Fig. 4).

The Danxia landforms of the Southeast China region span from central Zhejiang province to the southwest of Guangxi Province with north-east-trending ribbon formations, mainly concentrated in the joint border of Jiangxi Province and Fujian Province. The Danxia landforms of the Sichuan Basin region are mainly located in the southern edge of Sichuan Basin. The Danxia landfoms of the Qilian-Liupan region are mainly through the border of the Qilian fold system, the Liupanshan fold system and the Erdos Basin.

**Figure 5:**
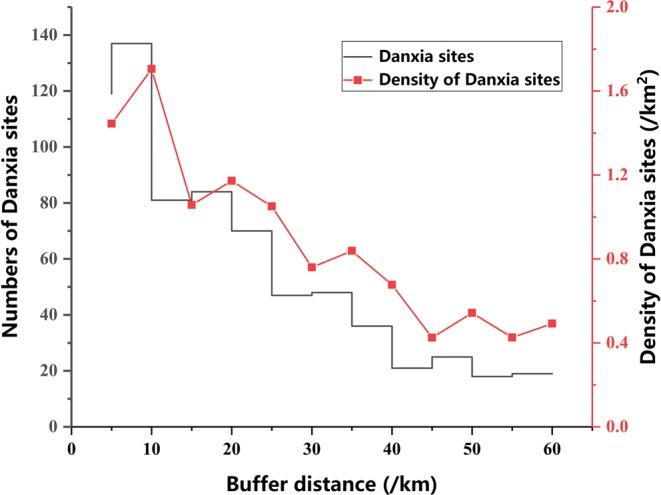
The trend of numbers and density of Danxia landforms with the increase of distance of buffer from fault.

The overlay analysis map of distributions of Danxia landforms and faults shows that in the 0–20 km fault region, there are 421 Danxia sites, accounting for 38.3% of the total number of Danxia sites; in the 20–40 km fault region, there are 201 Danxia sites, accounting for 18.2% of the total number of Danxia sites; in the 40–60 km fault region, there are 83 Danxia sites, accounting for 7.6% of the total number of Danxia sites (Fig. 5). Considering the different areas of buffer zones, the density of Danxia sites in different buffer zones was calculated. The result shows that the density of Danxia sites decreases from 0.025/km^2^ in 0–5 km fault region, 0.029/km^2^ in 5–10 km fault region, to 0.008/km^2^ in 50–55 km fault region, 0.010/km^2^ in 55–60 km fault region (Fig. 5). On the whole, the number and density of Danxia sites has a negative correlation with any given site’s distance from a fault. This indicates that faults are an important and influential factor for Danxia landform development.

### The distribution features of red beds on different tectonic units

**Figure 6:**
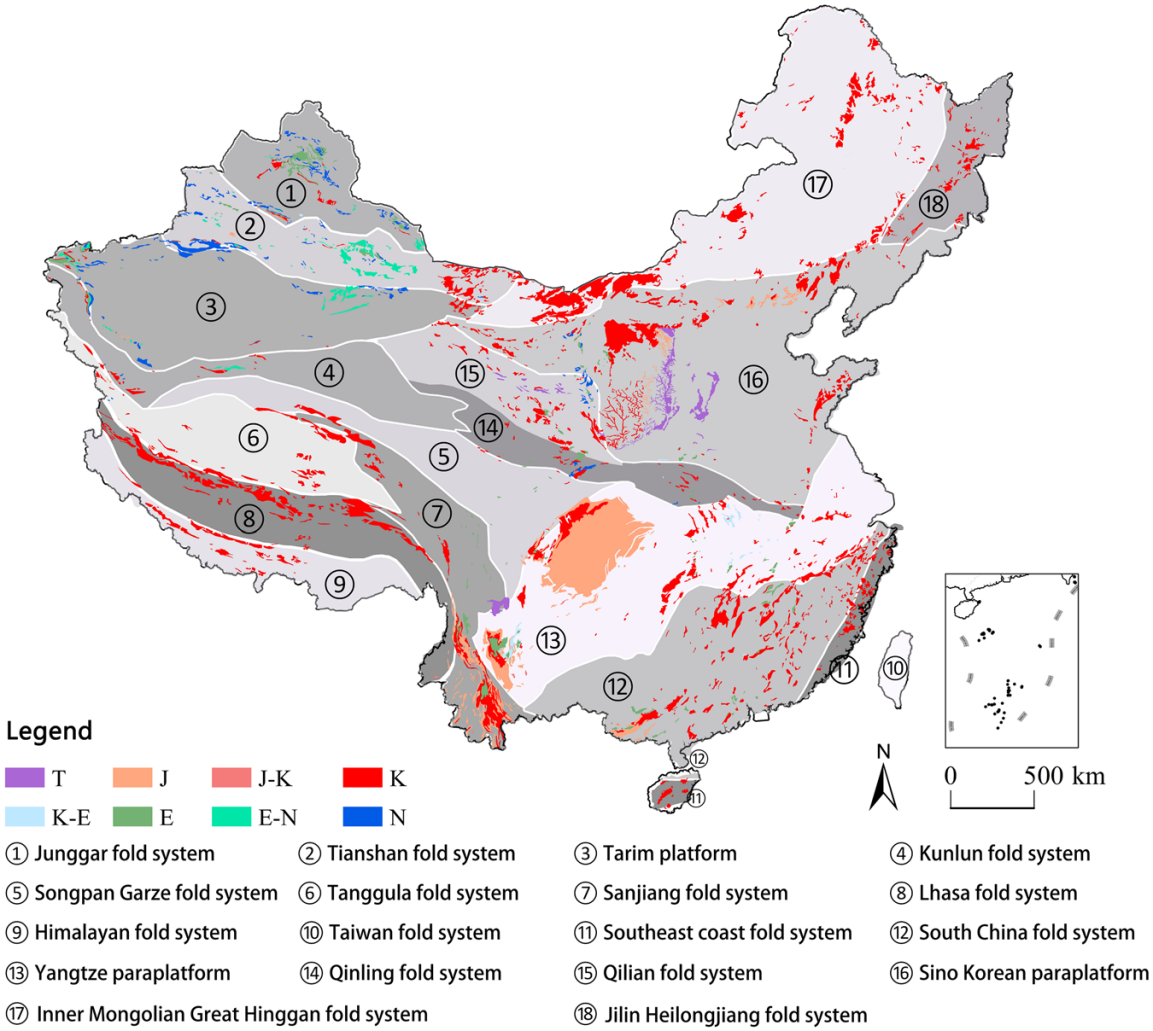
The distribution of red beds, differing tectonics produced by authors using ArcGIS 10.2 (http://www.esri.com/software/arcgis/arcgis-for-desktop). The red bed layer and tectonic layer were identified from77 National Geological maps (1:1,000,000) and the National Plate Tectonics Map (based on Plate Tectonic Theory) (1:3,200,000) 17, respectively. T-Triassic, J-Jurassic, K-Cretaceous, E-Paleogene, N-Neogene.

In order to analyze the underlying geologic factors that affect the distribution pattern of Danxia sites, folding systems and paraplatforms are included. By overlaying the tectonics of China on the distribution of red beds (Fig. 6), what can be found is that the stratigraphic age of red beds is older in paraplatform areas, and newer red bed stratum are in fold systems. As for areas of the red basins, red basins are larger in paraplatform areas, for example, Sichuan Basin in the Yangtze paraplatform, Ordos Basin in the Sino Korean paraplatform. Moreover, the distribution of red beds is patchy in fold systems.

## Tectonic Influences On the Distribution of Red Beds and Danxia Landforms

### Tectonic controls over the development of Danxia Landforms

#### The controlling effect of a regional structure on a sedimentary basin

From the Mesozoic onward, strong tectonic activation took place in many platforms that had been stable after the Hercynian orogeny in China. In eastern China, a series of NE-striking and NNE-striking upwarping zones and down-warping zones were formed under the influence of Pacific Plate activities. In the western area, land emerged gradually southward and some basins were formed under the influence of the India Plate activities. In central China, however, a NE-striking compression-shear belt took shape. Therefore, the Mesozoic and Cenozoic basins are distributed basically in similar pattern, which controls the distribution of the red basins.

#### The controlling effect of vertical movement of the Earth’s crust on the development processes of Danxia landforms

Red basins must be located in an uplifting area in late stage development in order to provide the necessary conditions for erosion to take place. After uplifting to a certain level, the crust must maintain relative stability for a sufficiently long period to facilitate Danxia landform to evolve gradually and continuously form early to late stages. Intermittent uplifting, however, can give rise to multi-terraced Danxia landforms. In Qiyunshan of Anhui Province, for example, this kind of alternating steep and gentle slopes association has as many as five terraces. According to thermoluminescent dating used on samples of alluvium from the river terrace of Mt. Danxiashan area by Huangjin, Liu Shangren *et al*.(1994)^32^, the Earth’s crust that area has been uplifted at a rate of 0.97 m/10 ka on average. Hence, the recent landform of Mt. Danxiashan was formed at about 6 Ma, while the red escarpment retreats at a rate of about 0.5–0.7 m/10 ka on average.

Owing to the outstanding landscape value, Danxia landform is a matter of great concern for geomorpologists. However, Danxia landform is just one type of the landforms developed from red beds. Red beds are not destined to develop into Danxia landform^33^. On the margin of a red basin are often accumulated pluvial chaotic mud gravels of tremendous thickness, which gradate toward the center into pluvial-alluvial conglomerate and sandy conglomerate, and fluvial-lacustrine sandstone, siltstone or politic rocks^34^. Danxia landform has generally developed from the conglomerates and sandstones which were mostly deposited in a basin fringe^35^. In the central area of a large basin, due to weak structural influences and weathering-resistance, only gentle red bed hills have developed.

### Tectonic impacts on the distribution of red beds and Danxia landforms

#### The distribution of red beds in basins formed during different stages of crustal movements

**Figure 7:**
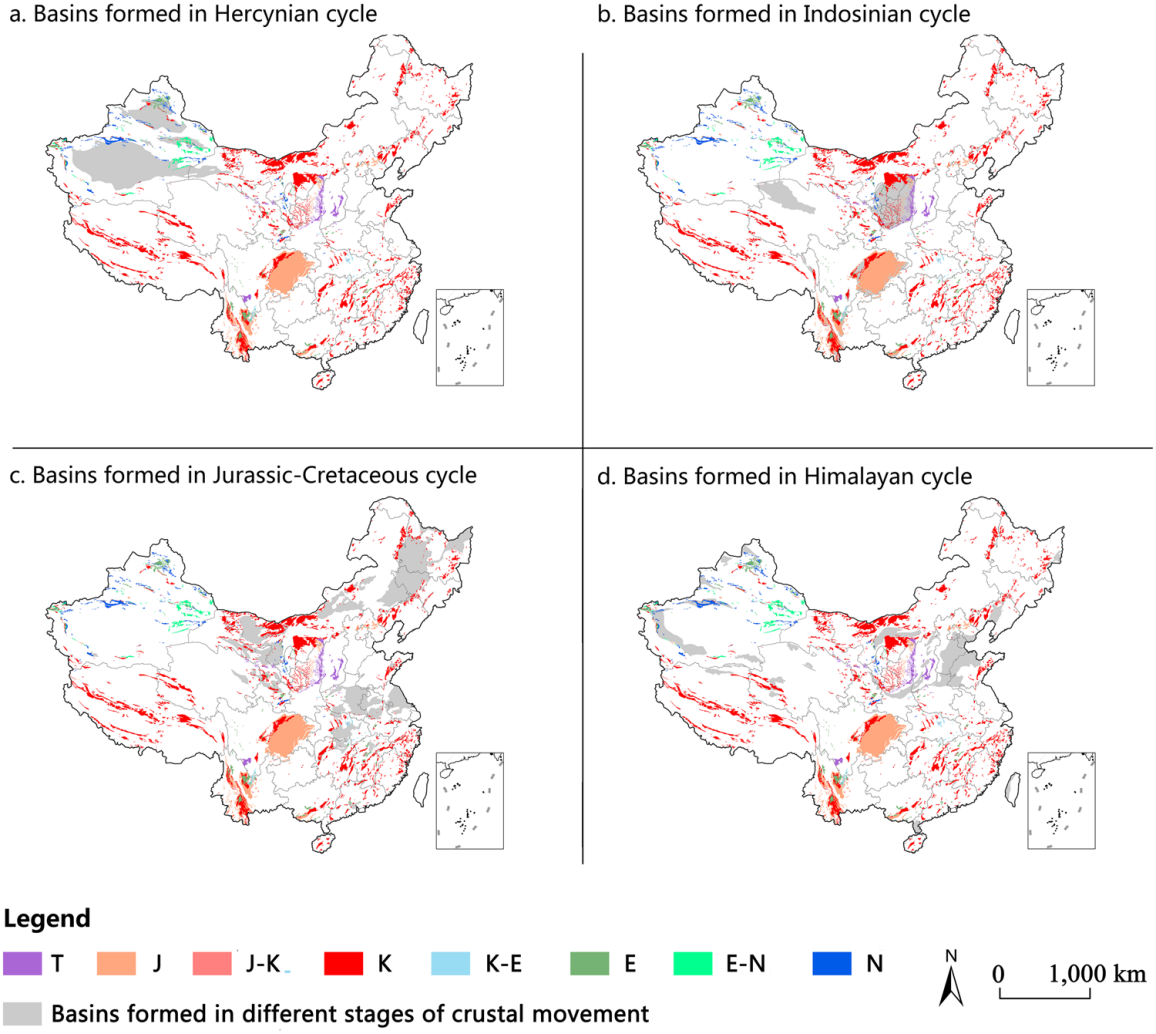
The distribution of red beds in basins formed during different stages of crustal movements. The red bed layer was identified from 77 National Geological maps (1:1,000,000) and the basins formed in different stages of crustal movements were extracted from the National Geotectonic Map produced by the Chinese Academy of Geological Sciences, then two layers were overlapped on ArcGIS 10.2 (http://www.esri.com/software/arcgis/arcgis-for-desktop). T-Triassic, J-Jurassic, K-Cretaceous, E-Paleogene, N-Neogene.

Red beds, as specific historical products of crustal evolution, are affected by multiple geological factors, and are especially affected by tectonic orogenies. Therefore, the overlay analysis map was carried out on the distribution of red beds and basins formed during different stages of crustal movement (Fig. 7).

The Indosinian cycle, Jurassic-Cretaceous cycle and Himalaya cycle had huge impacts on the tectonic framework in China, and were also the main stages in which the sediment that makes up red beds was deposited. The results show that most of the Triassic and Jurassic red beds in basins are distributed in Indosinian basins. In Jurassic-Cretaceous basins, the red beds mainly consist of Cretaceous and Paleogene red bed deposits. In Himalaya basins, most of the Neogene red beds were deposited. In basins formed in the Hercynian cycle, basically no exposed red beds can be found. The Indosinian cycle from the middle Permian to the end of upper Triassic controlled the deposition of Triassic, Jurassic and part of the Cretaceous red beds. The Jurassic-Cretaceous cycle from the beginning of the lower Jurassic to the end of the lower Cretaceous controlled the deposition of Cretaceous and Paleogene red beds. The Himalaya cycle from the beginning of the Cretaceous to the present controlled the deposition of Neogene red beds.

#### The impact of crustal movement on the exposure and distribution of red beds

According to the current distribution of red beds, the long-term geodynamic context, original distribution was considered by discussing the impact of crustal movement on the exposure and distribution of red beds.

The Junggar and Tarim basins were formed during Hercynian cycle^36^. From the Triassic, the Tarim Basin started its evolution stage as a continental basin^37^, and as a result, only a small area of marine red beds can be found in underlying formation. At the end of the Triassic, the collision between the Qiangtang plate and the Tarim plate led to this region being surrounded by mountains and red bed sediment deposition. Afterward, from the Cretaceous to the Paleogene, red bed material was deposited in the small basins on the fringe of Tarim Basin, which were formed during Himalaya cycles^36^. Although, outcrops of red beds can seldom be found in Junggar Basin, borehole data shows over 1,000 m of Middle Jurassic and Cretaceous red beds deposited there^38^.

During the Indosinian cycle (Fig. 7b), three large basins formed, namely, Ordos Basin, Sichuan Basin, and Qaidam Basin^39^. In the Upper Jurassic and the Cretaceous, a set of continental red bed material was deposited in Qaidam with a thickness of 448m^40^. Ordos Basin, the second largest sedimentary basin^41^, started at the inland lake basin stage as the basin margin uplifted, red bed material was deposited^42^. Due to relatively fast uplifting during the Himalayan cycle^43,44^, red beds in Sichuan Basin are exposed above the surface. Although Ordos Basin also uplifted rather quickly during the Himalayan cycle^45^, red beds are only exposed in deep ravines due to the deep loess cover. Due to continuous subsiding from the Lower Jurassic to the Eocene, and limited uplifting afterward^46^, red beds in Qaidam are not exposed.

During the Jurassic-Cretaceous cycle (Fig. 7c), Jurassic and Cretaceous red bed material deposited in the Hefei Basin and Subei Basin in East China, and most of this material was buried by Quaternary sediments^47^. Songliao Basin also had a wide distribution of Upper Cretaceous red beds during the Jurassic-Cretaceous cycle^48^, however, due to extensional tectonics in East China during the Himalayan cycle, uplifting occurred continuously in Songliao Basin and the basin eventually subsided^36^, causing most of the red beds to be covered by Quaternary sediment except the red beds in the boundary of the basin. In Southeast China, the Late Cretaceous red bed materials were mostly deposited in a dustpan-like half-graben under a back-arc extension regime when Southeast China was possibly influenced by northwestward subduction of the Palaeo-Pacific plate beneath East Asia^49^, because of intermittent uplifting since the Neogene^50^, and most of the red beds are exposed. In addition, during the third act of the Jurassic-Cretaceous cycle after the Early Cretaceous, the folding up of many small scale basins, formed during the Jurassic period in East China^51^, might have caused complete erosion of most Jurassic red beds and may explain why Jurassic red beds are seldom found. Whereas with small areas of red beds older than Jurassic in age, for example, Early Late Carboniferous red beds in eastern Liaoning Province^52^, what can be inferred is the possibility that most of the pre-Mesozoic red beds were completely eroded during subsequent mountain building periods.

Bohai Bay Basin formed during Himalayan cycle (Fig. 7d) and deposited an Eocene series of red bed material^53^. Since the Neogene, Bohai Bay Basin has transformed into a regional depression deposit^36^, therefore, there are no outcrops of red beds in this region. In this period, basins in West China, such as the Tarim, Junggar, and Qaidam Basins, which are associated with north-directed compression and exhibit thrust movements and flexural subsidence along basin margins^36^, had Paleogene and Neogene red bed material deposited within small basins nearby these larger basins, especially in the Xinjiang Uygur Autonomous region.

By analyzing the current distribution of red beds in large basins, Current distribution of red beds reflects the combining effect of crustal uplift afterward and the original deposition of red beds in basins. Red beds were found in most of the large basins formed in different geological period, however, as subsiding or limited uplifting of basins, most of red beds did not widely exposed except Sichuan Basin. Meanwhile, older red basins (pre-Cretaceous) may have fallen victims to erosion during subsequent mountain building periods.

#### The impact of the Himalayan cycle on the distribution of Danxia landforms

As presented in 3.2, three concentrated Danxia sites are recognized, namely the Southeast China region, the Sichuan Basin region and the Qilian-Liupan region. Landform is the combined result of crustal uplifting and denudation, the distribution of Danxia landforms in China is analyzed as follows:

During the Jurassic-Cretaceous cycle, red beds material was deposited in fault basins in Southeast China. Since the Neogene, most part of South China uplifted intermittently and large areas of mountains started being impacted by denudation and erosion, leading to various landscapes being formed^50^, including Danxia landforms.

Since the Upper Cretaceous, the crustal movement around Sichuan Basin experienced three periods. In the first period, most of this region rifted except for the western part, which subsided and a small area of Paleogene red bed materials were deposited. During the second period, the whole basin uplifted up 1 km at a speed of 40 m/Ma. During the third period, most of the Sichuan Basin uplifted 2.5 km at a speed of 100 m/Ma^43,44^. On the whole, the main rifting of Sichuan Basin occurred in the Neogene, the whole basin rifted 4.2 km, the speed of rifting was over 100 m/Ma^44^. However, the denudation height in Sichuan Basin is 2–3 km on average. In this geological setting, Sichuan Basin became one of the regions of Danxia landform concenration.

Since the Himalayan Movement, the regional tectonics have been largely changed by faulting. Marginal thrusting has also played an important role, especially in the early stage, Mt. Qilianshan and Mt. Liupanshan might have been built mostly in the Quaternary^54^. Rifting occurred in the Qilian-Liupan region between 8.1MaBP to 3.8 MaBP, this region uplifted 750 m at a speed of 197 m/Ma^55^.

In addition, although the Ordos Basin experienced intermittent uplift from the Early Pleistocene to the Holocene during the Himalayan cycle^45^, Danxia landforms were not presented in great numbers because the short period of denudation and red beds were covered by huge thick loess.

The Himalayan Movement played a key role in the formation of Danxia landform. Theoretically, the Crustal cycles before Himalayan Movement might also cause the fast uplifting of red basin, and then the aboveground red beds could form Danxia landform under the function of external agency. However, after a long historical period, most of them had fallen victims to erosion.

## Conclusion

The exposure of red beds covers an area of 9.16 × 10^5^ km^2^, which accounts for 9.5% of the total land area of China. Red beds were deposited from the Triassic to the Neogene. Among them, the Cretaceous red beds account for 57% of the total area of red beds, Jurassic for 25%, Neogene for 4.8%, Paleogene for 5.1%, and Triassic for 4%. The Triassic red beds are mainly distributed in the east of the Ordos Basin and exposed in strong cutting gullies with dendritic distributions. Jurassic red beds are mainly distributed in Sichuan Basin, Cretaceous red beds are distributed across the entire country, with the exception of the Xinjiang Uygur Autonomous Region. Paleogene and Neogene red beds are mainly distributed in the margin of Tarim Basin and Junggar Basin, Xinjiang Uygur Autonomous Region. The area and proportion of Paleogene and Cretaceous red beds have a negative correlation with the distance to a fault, indicating that faults are an important controlling factor for Cretaceous and Paleogene red beds.

The spatial distribution of Danxia landforms is highly clustered, three zones are identified: Southeast China region, the Sichuan Basin region, and the Qilian-Liupan region. The number and density of Danxia sites also have a negative correlation with distance from a fault, again stressing that faults are an important controlling factor for Danxia landform development. The development of Danxia landforms is controlled by the tectonic controls over the red beds and the vertical movement of the Earth’s crust.

In paraplatform areas, the stratigraphic age of red beds is relatively older than that in fold systems, and the area of red basins is larger in paraplatforms than that in fold systems. Buried red beds can be found in most large basins formed in different cycles of crustal movement, however, due to the basin’s subsiding or limited uplifting, most of them have not been widely exposed except in Sichuan Basin. Alternatively, older red basins (pre-Cretaceous) may have been eroded during subsequent mountain building periods.

The Himalayan Movement played a key role in the distribution of Danxia landforms by causing fast crustal uplifting in red beds concentrated areas.
